# Brain inflammation and oxidative stress in a transgenic mouse model of Alzheimer-like brain amyloidosis

**DOI:** 10.1186/1742-2094-1-21

**Published:** 2004-10-22

**Authors:** Yuemang Yao, Cinzia Chinnici, Hanguan Tang, John Q Trojanowski, Virginia MY Lee, Domenico Praticò

**Affiliations:** 1 Center for Experimental Therapeutics and Department of Pharmacology; University of Pennsylvania, School of Medicine, Philadelphia, PA 19104 USA; 2 Center for Neurodegenerative Disease Research; University of Pennsylvania, School of Medicine, Philadelphia, PA 19104 USA; 3 Institute on Aging; University of Pennsylvania, School of Medicine, Philadelphia, PA 19104 USA

## Abstract

**Background:**

An increasing body of evidence implicates both brain inflammation and oxidative stress in the pathogenesis of Alzheimer's disease (AD). The relevance of their interaction in vivo, however, is unknown. Previously, we have shown that separate pharmacological targeting of these two components results in amelioration of the amyloidogenic phenotype of a transgenic mouse model of AD-like brain amyloidosis (Tg2576).

**Methods:**

In the present study, we investigated the therapeutic effects of a combination of an anti-inflammatory agent, indomethacin, and a natural anti-oxidant, vitamin E, in the Tg2576 mice. For this reason, animals were treated continuously from 8 (prior to Aβ deposition) through 15 (when Aβ deposits are abundant) months of age.

**Results:**

At the end of the study, these therapeutic interventions suppressed brain inflammatory and oxidative stress responses in the mice. This effect was accompanied by significant reductions of soluble and insoluble Aβ1-40 and Aβ1-42 in neocortex and hippocampus, wherein the burden of Aβ deposits also was significantly decreased.

**Conclusions:**

The results of the present study support the concept that brain oxidative stress and inflammation coexist in this animal model of AD-like brain amyloidosis, but they represent two distinct therapeutic targets in the disease pathogenesis. We propose that a combination of anti-inflammatory and anti-oxidant drugs may be a useful strategy for treating AD.

## Introduction

Alzheimer's disease (AD) is the most common, complex and challenging form of neurodegenerative disease associated with dementia in the elderly. Neuropathological examination of the AD brain shows extensive neuronal loss, accumulation of fibrillar proteins as extra-cellular amyloid β (Aβ) plaques, and as neurofibrillary tangles (NFTs) inside neurons [1]. However, besides these pathological hallmarks, AD brains exhibit clear evidence of chronic inflammation and oxidative damage [2,3]. Currently, data from human studies as well as animal models strongly support the concept that oxidative imbalance and subsequent oxidative stress are among the earliest events in the pathogenesis of AD [4,5]. Thus, an increase in lipid peroxidation, protein oxidation and DNA oxidation has been reported not only in AD patients, but also in subjects with mild cognitive impairment (MCI) [6,7]. Similarly, immunohistochemical and biochemical evidence for these signatures of oxidative stress have been shown in animal models of AD-like brain amyloidosis, i.e. the Tg2576 transgenic mouse model thereof [8-10]. Chronic neuroinflammation is another constant feature of AD, and this also is thought to play a significant role in the onset and progression of AD. Support for this hypothesis comes from epidemiological studies showing that prolonged use of nonsteroidal anti-inflammatory drugs (NSAIDs) decreases the risk of developing AD as well as delaying the onset of this disorder [11], while many mediators of inflammation have been detected in the AD brain [12]. Further, recent studies in AD mouse models have shown that chronic treatment with a subset of NSAIDs (e.g. ibuprofen, flurbiprofen, indomethacin) reduced brain inflammation and Aβ levels in addition to the deposition of Aβ in brain [13,14]. Despite this evidence, and the considerable theoretical and therapeutic interest, the relationship between brain inflammation responses and oxidative stress has not yet been clearly delineated in AD. For example, its is possible to consider these two events as elements of the same response mechanism, or they can be envisaged as two separate events. Alternatively, they also could work in concert to contribute synergistically to the pathogenesis of AD.

In the present study, we examined whether the simultaneous administration of an anti-oxidant, vitamin E, with an anti-inflammatory drug, indomethacin, would exert an additive anti-amyloidogenic effect in the Tg2576 mouse model of AD-like Aβ brain amyloidosis, one of the most extensively studied mouse models of AD [15]. Significantly, we found for the first time that coincidental suppression of brain oxidative stress further augments the anti-amylodogenic effect of indomethacin.

## Materials and Methods

### Animals

The genotype and phenotype features of the heterozygote Tg2576 mice that we studied here have been described in earlier reports on these mice from our group [9]. Mice were weaned at 4 weeks, kept on a chow diet, and males were always separated from females for the entire study. Eight-month-old Tg animals were divided in two groups (n = 10 each), and randomized to receive placebo, or simultaneously indomethacin (10 mg/liter) in their drinking water, and vitamin E (α-tocopherol) in their diet (2 I.U./mg diet) for seven months before being sacrificed. The detailed dosing of the animals receiving indomethacin or vitamin E alone (at the same concentration used in the present study) were described in two previously published studies which also included data on numerous non-transgenenic littermate controls of the Tg2576 mice [16,17]. Fresh drinking water and diet were always replaced every other day. Preliminary experiments demonstrated that the selected dose of indomethacin suppressed total cylcooxygenase-1 activity *in vivo *and significantly reduced brain inflammation [16]. The high dose of vitamin E was selected based on a previous study, which indicated that at this concentration it significantly reduced brain oxidative stress response [17]. During the study, all mice gained weight regularly, and no significant difference was detected between the two groups.

### Tissue preparation

Animals were anesthetized and euthanized following procedures recommended by the Panel on Euthanasia of the American Veterinary Medical Association. They were always perfused intra-cardially for 30 min with ice-cold 0.9% phosphate buffer saline (PBS), containing EDTA (2 mM/L) and BHT (2 mM/L), pH7.4. Brains were removed and one hemisphere was fixed by immersion in 4% paraformaldehyde in 0.1 M PBS (pH7.4) at 4°C overnight, blocked in the coronal plane, and embedded in paraffin as previously described for immunohistochemistry [9,16,17], The other hemisphere was gently rinsed in cold 0.9% PBS, then immediately dissected in three anatomical regions (total cerebral cortex, hippocampus, and cerebellum) for biochemistry.

### Biochemical analysis

Tissue samples were minced and homogenized, and total lipid extracted with ice-cold Folch solution (chloroform: methanol; 2:1, vol/vol). Lipids were subjected to base hydrolysis by adding aqueous 15% KOH and then incubated at 45°C for 1 hr for measurement of total iPF_2α_-VI by ion chemical ionization gas chromatography/mass spectrometry assay, as previously described [9,16,17]. In brief, a known amount of the internal standard is added to each sample, after solid phase extraction samples are derivatized and purified by thin layer chromatography, and finally analyzed. An aliquot of these extracts was assayed for total levels of PGE_2 _and TxB_2 _by a standardized ELISA kit following the manufacturer's instructions (Cayman Chem. Com.). Briefly, extracts were diluted with acetate buffer and purified through an affinity column. The purified samples were evaporated, re-dissolved in the assay buffer and applied to 96-well plates pre-coated with goat anti-serum IgG and incubated with PGE_2 _or TxB_2 _monoclonal antibodies. The plates were rinsed with washing buffer and developed using Ellman's reagent for 60–90 min at room temperature with gentle shaking. Specific concentrations were determined spectrophotometrically and expressed as pg/mg tissue.

IL-1β levels were measured by a standardized sandwich ELISA kit following the manufacturer's instructions (Endogen Pierce). Briefly, equal amounts of sample were loaded onto 96-well plate pre-coated with monoclonal antibody against mouse IL-1β overnight at 4°C. The plates were rinsed three times with washing buffer and developed with streptavidin-horseradish peroxidase (HRP) [13]. Specific concentrations were determined spectrophotometrically and expressed as pg/mg protein.

Total protein carbonyls in tissue were determined by using the Zenith PC test kit according to the manufacturer's instructions (Zenith Tech.) [18]. Briefly, aliquots of the tissue homogenates were first reacted with dinitrophenylhydrazine (DNP), transferred to a multi-well plate, incubated with blocking reagent, washed and probed with anti-DNP-biotin solution. After washing, samples were incubated with streptavidin-HRP, washed again, and then developed. After 15 min, the reaction was stopped and absorbance immediately read at 450 nm. Oxidized protein standards, internal controls and blanks were always assayed at the same time and in the same way. All samples were always determined in triplicate and in a blind fashion.

### Immunoblot analysis

An aliquot of brain homogenates was electrophoresed on a 10% acrylamide gel under reducing conditions. Protein were transferred to a polyvinylidene membrane before blocking in 10% nonfat dry milk for 2 hr. Blots were incubated with monoclonal antiboby against glial fibrillary acidic protein (GFAP) (2.2B10) (1:1,000), or an anti-beta actin (1:5,000) antibody overnight at 4°C. After three rinses, blots were incubated with HRP-conjugated goat anti-mouse for 45 min before development with chemiluminescent detection system using ECL (Amersham). Bands were quantified using densitometric software (Molecular Analyst). The anti-GFAP is a monoclonal antibody, and its characterization has previously been published [19] (Zymed Lab. Inc.). The anti-beta actin is from commercial sources (Novus Biological).

### Brain Aβ1-40 and Aβ1-42 levels

Sequential extraction of brain samples was performed with high-salt buffer and formic acid, respectively to measure soluble and insoluble Aβ1-40 and Aβ1-42 levels, as previously described [9,16,17]. Briefly, cerebral cortex, hippocampus and cerebellum were serially extracted in high-salt Re-assembly buffer (0.1 M Tris, 1 mM EGTA, 0.5 nM Mgso4, 0.75 M NaCl, and 0.02 M NaF, pH 7.0) containing protease inhibitor mixture (pepstatin A, leupeptin, N-tosyl-L-phenylalanine chloromethyl ketone, soybean trypsin inhibitor, each at l μg/ml in 5 mM EDTA). Homogenates were centrifuged at 100,000 × g for 1 hr at 4°C. Supernatants were removed, pellets were re-suspended in 70% formic acid and sonicated and centrifuged at 100,000 × g for 1 hr at 4°C. Supernatants were diluted 1:20 with 1 M Tris base. Samples were mixed with buffer EC [0.02 M sodium Phosphate, 0.2 mM EDTA, 0.4 M NaCl, 0.2% BSA, 0.05% CHAPS, 0.4% Block-ace (Dainippon, Suita, Osaka, Japan), 0.05% sodium azide, pH7.0] and analyzed directly using Ban 50/BA27 for Aβ1-40 or Ban50/BC-05 for Aβ1-42/43 sandwich ELISA system as previously described [16,17]. Results were expressed as pmol/g tissue. The values were calculated by comparison with a standard curve of synthetic Aβ1-40 and Aβ1-42. Analyses were always performed in duplicates and in a coded fashion.

### Burden of brain Aβ deposits

Serial 6-μm-thick paraffin sections were cut throughout each brain, and mounted on APES-coated slides. Sections were deparaffmized, hydrated, rinsed with PBS and pre-treated with formic acid (88%) for 10 min to antigen retrieval, and with 3% H_2_0_2 _in methanol for 30 min to eliminate endogenous peroxidase activity in the tissue and with the blocking solution (5% normal horse serum in Tris buffer, pH 7.6). Subsequently, sections were incubated with a biotinylated antibody against Aβ (4G8) (1:10,000 dilution), at 4°C overnight [16,17]. Sections were then incubated with secondary antibody for 1 hr (dilution 1:1,000), then reacted with horse-peroxidase-avidin-biotin complex (Vector Lab.), and immuncomplexes visualized by using 3,3'-diaminobenzidine as the chromogen. Finally, they were dehydrated with ethanol, cleared with xylene and coversliped with Cytoseal. As control, sections from the same group of animals were treated in the same manner, except for the primary antibody. Light microscopic images from the somatosensory cortex, perihippocampal cortex, and hippocampus were captured from eight series of sections using a Nikon Microphot-FXA microscope with 4 × objective lens. The area occupied by Aβ-immunoreactive products in the region of interest were identified, and the total area occupied by the outlined structures was measured to calculate: 1) the total area with selected immunoreactive products, 2) the percentage of the area occupied by immunoreactive products over the outlined anatomical area in the image, as previously described [9,16,17]. Analyses were always performed in a coded fashion.

### Statistical analysis

Data are expressed as mean ± standard error of mean (S.E.M.), analyzed by analysis of variance (ANOVA), and subsequently by student unpaired 2-tailed *t *test corrected for multiple comparisons. Significance was set at p < 0.05.

## Results

**Figure 1:**
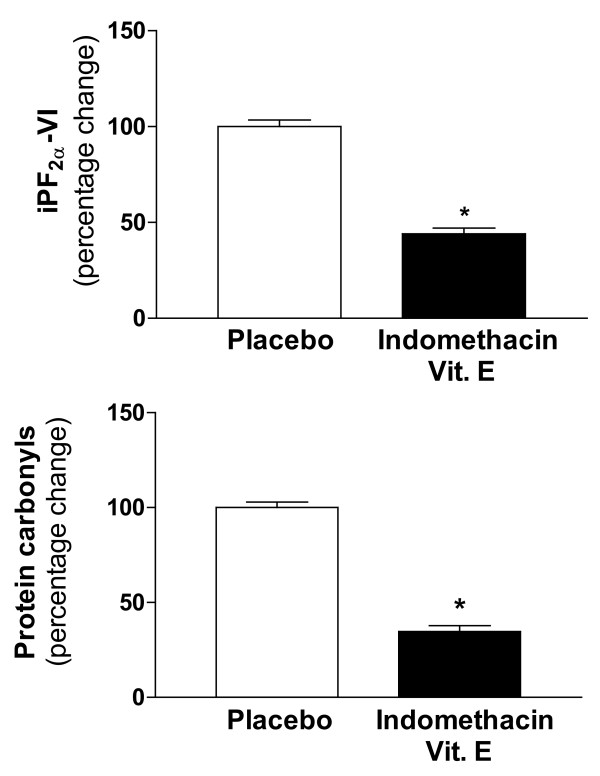
Effect of indomethacin plus vitamin E supplementation on markers of brain oxidative stress. Total cerebral cortex homogenates from Tg2576 receiving placebo (open bars) or the combination therapy (closed bars) were assayed for levels of iPF_2α_-VI (upper panel) and protein carbonyls (lower panel) (*p < 0.01, n = 10 per group).

Starting at eight months of age, Tg2576 mice were randomized to receive placebo or vitamin E (2 I.U./mg diet) added to their diet, plus indomethacin (l0 mg/liter) in their water, and they were treated until they were 15 months old. Notably, at 8 months of age, the Tg2576 mice show elevated brain levels of soluble and insoluble Aβ as well as isoprostanes, relative to their non-transgenic littermates, but they show no evidence of any brain Aβ deposits, while following the initial onset of mature plaque-like brain deposits at about 11–12 months of age, the Tg2576 mice show abundant Aβ deposits and higher levels of isoprostanes in neocortex and hippocampus a 15 months of age [9,15,20]. Assuming that each mouse eats 4–5 mg chow/day, the estimated average vitamin E intake for each animal was ~8–10 I.U./day. Assuming that each mouse drinks 3 to 4 mL water/day, the estimated daily intake of indomethacin was calculated around 30–40 ng. At the end of the study, body weight, total plasma cholesterol, triglycerides and peripheral blood cell count were not different between placebo and active treatment (not shown). Compared with placebo, Tg2576 mice receiving indomethacin plus vitamin E at the same time had a significant reduction in PGE_2 _and suppression of TxB_2 _levels in tissue homogenates from total cortex and hippocampus (Table 1). Further, the presence of vitamin E significantly reduced two independent markers of oxidative stress injury in both brain regions. Thus, neocortex and hippocampus levels of iPF_2α_-VI (a reliable biomarker of lipid peroxidation), as well as protein carbonyls (known biomarkers of protein oxidation) were both significantly decreased (Figure 1). Compliance with the diet was evident from the rise in brain levels of vitamin E (+57%) in the mice receiving the supplemented chow.

**Table 1:** Effects of indomethacin plus vitamin E on total brain cortex levels of PGE_2_, TxB_2 _and IL-1β in Tg2576 mice. Mice were treated starting at 8-months of age until they were 15-month-old (n = 10 animals per group).

	**Placebo**	**Indomethacin Vitamin E**	**P**
PGE_2 _(pg/mg tissue)	92 ± 8	39 ± 5*	<0.01
TxB_2 _(pg/mg tissue)	148 ± 10	15 ± 4*	<0.001
IL-1β (pg/mg protein)	75 ± 12	33 ± 8*	<0.01

Values are expressed as means ± S.E.M.

**Figure 2:**
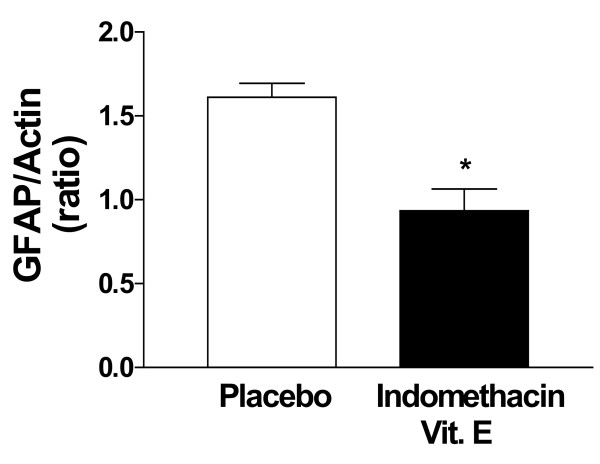
Effect of indomethacin plus vitamin E supplementation on GFAP levels. GFAP and actin levels were detected by immunoblots in homogenates from total cortex of Tg2576 administered with placebo (open bars) or indomethacin plus vitamin E (closed bars) (*p < 0.02, n = 8 per group).

Western blot analysis was used to determine the effect of the drug treatment on GFAP levels, a marker of astrocytosis [13]. These levels were significantly lower in the treated than in the placebo group (Figure 2). Another marker of brain inflammation was also assessed, i.e. IL-1β, which has been reported to be increased in these mice [13]. Compared with placebo, we found that IL-1β levels were significantly reduced by 55% in homogenates from neocortex (Table 1), and 61% in hippocampus (not shown) of the mice receiving the combination therapy.

**Figure 3:**
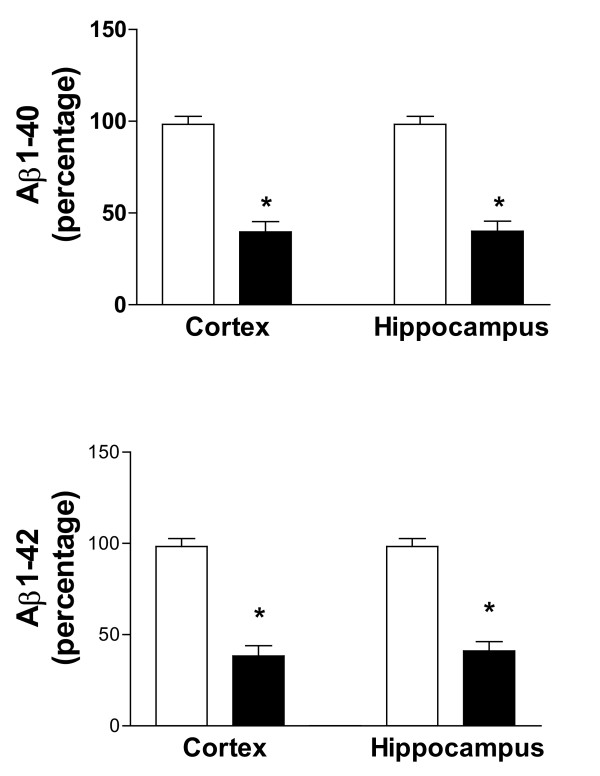
Effect of indomethacin plus vitamin E supplementation on soluble Aβ levels. Levels of high salt soluble Aβ1-40 and Aβ1-42 in total cortex and hippocampus of Tg2576 on placebo (open bars), or indomethacin plus vitamin E (closed bars) (*p < 0.01, n = 8 per group).

**Figure 4:**
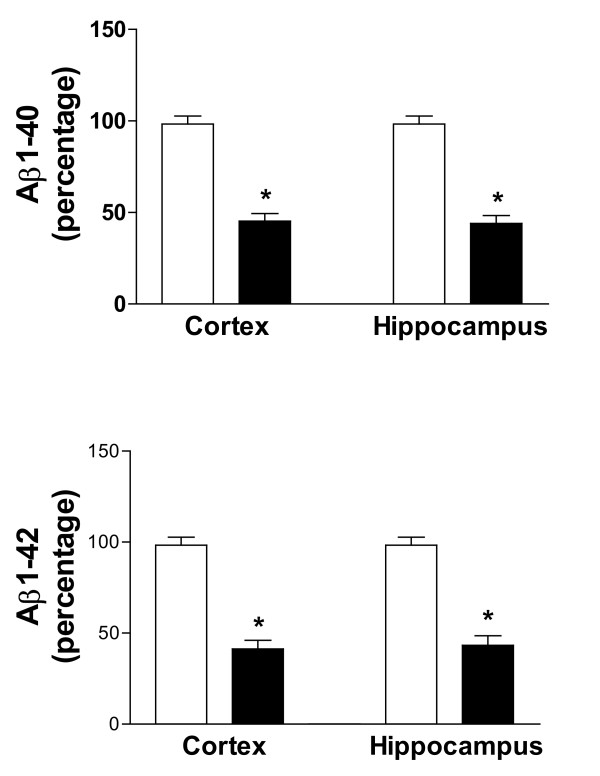
Effect of indomethacin plus vitamin E supplementation on insoluble Aβ levels. Levels of formic acid soluble Aβ1-40 and Aβ1-42 in total cortex and hippocampus homogenates from Tg2576 receiving placebo (open bars) or indomethacin plus vitamin E (closed bars) (*p < 0.001, n = 8 per group).

**Figure 5:**
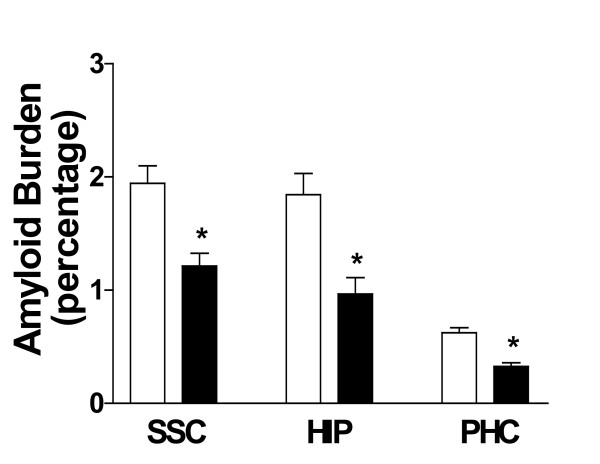
Effect of indomethacin plus vitamin E supplementation on amyloid deposition. Percentage area of somatosensory cortex (SSC), hippocampus (HIP) and parahippocampal cortex (PHC) occupied by Aβ immunoreactive deposits in Tg2576 receiving placebo (open bars), or indomethacin plus vitamin E (closed bars) for seven months (*p < 0.001; n = 8 per group).

**Figure 6:**
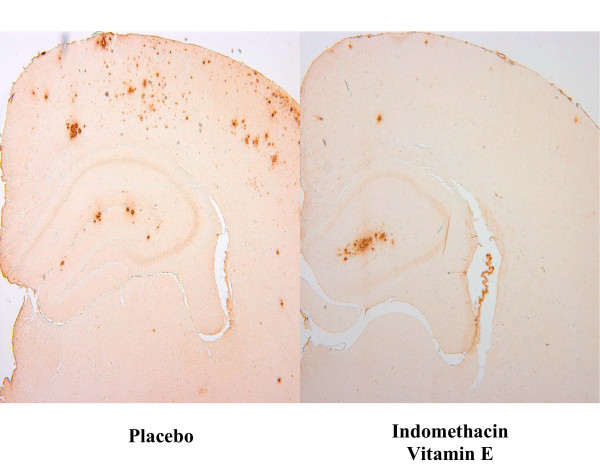
Representative pictures of brain sections from mice on placebo or receiving indomethacin plus vitamin E.

Next, we assessed the effect of indomethacin and vitamin E on brain levels of soluble and insoluble Aβ1-40 and Aβ1-42. As expected for their age, Tg2576 mice on placebo showed elevated levels of both forms of these peptides in their cortex and hippocampus (Figure 3), whereas cerebellum had much lower levels (not shown). Soluble Aβ1-40 and Aβ1-42 were reduced by ~65% in both neocortex and hippocampus homogenates from treated mice (Figure 3). Further, we found that the combination of vitamin E with indomethacin resulted in a significant reduction (55% and 59%) of the insoluble fraction of these peptides in both brain regions (Figure 4). The same treatment had no effect on both forms of Aβ in the cerebellum of Tg2576 compared with placebo (not shown). Amyloid deposits were widely present in the cerebral cortex and hippocampus of Tg2576 mice at 15 months of age, as previously reported [15,20]. To determine the effect of this treatment on amyloid deposition, the areas occupied by 4G8-immunopositive reactions were analyzed in three different brain regions: the somatosensory cortex (SSC), perihippocampal cortex (PHC), and hippocampus (HIP) areas. Comparison of the burden of Aβ positive deposits between placebo and combination therapy groups revealed a significant reduction for the amyloid burden in all three regions considered (Figure 5, 6).

## Discussion

There is substantial evidence implicating both oxidative stress and inflammatory mechanisms in AD pathogenesis. Evidence for oxidative stress derives from both human (post-mortem and living patients) studies, and transgenic mouse models of the disease. There is a long list of surrogate markers of reactive oxygen species-mediated injury that have been found increased in the brain and cerebrospinal fluid of AD patients. It includes, just to mention a few, malondialdehyde, 4-hydroxynonenal, F_2_-isoprostanes (lipid peroxidation); protein carbonyls, nitrotyrosine (protein oxdidation); 8-hydroxy-2'-deoxyguanosine (DNA oxidation) [3-5]. Transgenic animals show the same type of oxidative damage that is found in AD, and it directly correlates with the presence of Aβ deposits [8,10,21]. Oxidative stress also precedes amyloid deposition in human AD, the Tg2576 and a transgenic *Caernorhabditis elegans *model, which over-expresses Aβ1-42 [9,22,23]. Furthermore, dietary or genetic perturbation of the anti-oxidant defense system causes exacerbation of the amyloid pathology characteristic of Tg models [24,25]. Taken together, the data accumulated so far clearly indicate that oxidative imbalance and subsequent chronic oxidative stress are not only early events, but they also play a functional role in AD pathogenesis. Based on this evidence we started the treatment at an early stage before the amyloid deposition occurs.

Inflammatory mechanisms are also operative in the AD brain and significantly contribute to the pathophysiology of the disease. Although classical defined inflammation, including such features as edema and neutrophil invasion, is not seen in the AD brain, hallmark of innate immune response are constant elements of the neuropathology associated with brain degeneration in AD [12]. Further, evidence that inflammation contributes to the AD pathogenesis stems out from several retrospective epidemiological studies showing a significant reduction in the risk of AD associated with a prolonged usage of NSAIDs [11]. Tg2576 mice display age-related neocortical and hippocampal amyloid deposits, which correlate with microglia activation, reactive astrocytes with increased GFAP, IL-1β levels, and dystrophic neuritis [13,26]. Furthermore, plaque-associated reactive microglia in these animals show enhanced staining for TNFα and IL-1β [27].

Lim et al. first reported that chronic administration of the NSAID ibuprofen to Tg2576 reduces total Aβ levels, amyloid burden and brain inflammation [13]. More recently, we showed that a high dose of indomethacin, another NSAIDs member, which fully suppresses total cyclooxygenase (COX)-l activity, by modulating brain inflammation response reduces soluble Aβ1-40 and Aβ1-42, and insoluble Aβ1-42 but not Aβ1-40 levels in the same model. This effect was accompanied by a significant reduction of the amyloid burden in the hippocampus of these mice [16]. However, recent studies indicate that a subset of NSAIDs, including indomethacin, also possesses a direct, COX-independent Aβ-lowering capacity in cell cultures as well as transgenic models [28]. Further, we showed that vitamin E alone at the same high dose used in this study decreased soluble and insoluble Aβ1-40 and Aβ1-42 levels by ~28% and ~35%, respectively. This effect was associated with a significant reduction in amyloid deposition in the somatosensory cortex, but not in the hippocampus or parahippocampal areas [17].

In the present study, we extended these previous observations by examining whether administration of indomethacin in combination with vitamin E would result in a better anti-amyloidotic effect. Our findings show that soluble Aβ1-40 and Aβ1-42 levels were reduced by ~65%, while the insoluble fractions were decreased by ~55%. Consistently, we observed a better and more diffuse effect also on the amount of amyloid deposited in the brain at the end of the study. Finally, the two drugs together produced an additive affect on brain inflammation and oxidative stress [16,17].

Our results confirm previous observation where low-dose curcumin, a drug with reported both anti-oxidant and anti-inflammatory activities, reduced total Aβ and plaque burden [29]. However, several other mechanisms of action, unrelated to inflammation or oxidation, could underlie the effect of this compound in vivo, and the relative importance of each of them for the anti-amyloid effect observed is still unclear [30].

In our study, we used two different drugs with a more restricted therapeutic target to provide further evidence that both oxidative stress and inflammation are indeed functionally relevant in the development of the phenotype of these animals. However, we also provide new information on the critical issue of the in vivo relationship between these two events. Thus, our results suggest that brain inflammation and oxidative stress are two separate events, which work in concert to modulate the development of this AD-like brain Aβ amyloidosis model. Previously, we have shown that a full dose of indomethacin alone despite a significant reduction in brain inflammation had only a marginal effect on brain oxidative stress in the Tg2576 mice [16]. This finding suggests that lipid peroxidation products contribute minimally to brain inflammation in this model, and raise the possibility that vitamin E alone might have influenced amyloidosis by other mechanisms related to its anti-oxidant effect, such as inflammation. Thus, we observed that this antioxidant further suppressed both amyloidosis and brain inflammation when combined with indomethacin.

In summary, our findings support the hypothesis that oxidative stress and inflammation represent important but distinct therapeutic targets in AD-like amyloidosis. We conclude that a combination of therapeutic agents targeting these different disease-modulating mechanisms might be rationally evaluated in the prevention or therapy of AD in humans.

## List of abbreviations

AD: Alzheimer's disease

Aβ: Amyloid β peptide

Tg: Transgenic mouse model

NSAIDs: Non-steroidal anti-inflammatory drugs

PGE_2_: Prostaglandin E_2_

TxB_2_: Thromboxane A_2_

GFAP: Glial fibrillary acidic protein

IL-1β: Interleukin 1-β

IPF_2α_-VI: Isoprostane F_2α_-VI

## Competing interests

The authors declare that they have no competing interests.

## Authors' contributions

Yuemang Yao and Cinzia Chinnici have made substantial contribution to the acquisition of data and biochemical analyses. Hanguan Tang contributed to the immunohistochemical analyses. John Q. Trojanowski and Virginia M-Y Lee have been involved in the interpretation of data, and the critical revision of the manuscript for intellectual content. Domenico Praticò has been involved in the conception and design of the studies, interpretation of data, drafting and critical revision of the manuscript.
